# Mortality Predictors for Adult Patients with Mild-to-Moderate Traumatic Brain Injury: A Literature Review

**DOI:** 10.3390/neurolint16020030

**Published:** 2024-04-05

**Authors:** Ansam Eghzawi, Alameen Alsabbah, Shatha Gharaibeh, Iktimal Alwan, Abeer Gharaibeh, Anita V. Goyal

**Affiliations:** 1 Insight Research Institute, Flint, MI 48507, USA; ansam.eghzawi@iinn.com (A.E.); alameen.alsabbah@iinn.com (A.A.); shatha.gharaibeh@iinn.com (S.G.); iktimal.alwan@iinn.com (I.A.); 2 Center for Cognition and Neuroethics, University of Michigan-Flint, Flint, MI 48502, USA; 3 Department of Research, Insight Hospital and Medical Center, Chicago, IL 60616 USA; 4 Department of Emergency Medicine, Insight Hospital and Medical Center, Chicago, IL 60616, USA

**Keywords:** traumatic brain injury, TBI, mortality, trauma, head injury

## Abstract

Traumatic brain injuries (TBIs) represent a significant public health concern, with mild-to-moderate cases comprising a substantial portion of incidents. Understanding the predictors of mortality among adult patients with mild-to-moderate TBIs is crucial for optimizing clinical management and improving outcomes. This literature review examines the existing research to identify and analyze the mortality predictors in this patient population. Through a comprehensive review of peer-reviewed articles and clinical studies, key prognostic factors, such as age, Glasgow Coma Scale (GCS) score, the presence of intracranial hemorrhage, pupillary reactivity, and coexisting medical conditions, are explored. Additionally, this review investigates the role of advanced imaging modalities, biomarkers, and scoring systems in predicting mortality following a mild-to-moderate TBI. By synthesizing the findings from diverse studies, this review aims to provide clinicians and researchers with valuable insights into the factors influencing mortality outcomes in adult patients with a mild-to-moderate TBI, thus facilitating more informed decision making and targeted interventions in clinical practice.

## 1. Introduction

A traumatic brain injury (TBI) is defined as any damage to the head, scalp, or brain associated with an altered mental status or death caused by an external force and that can lead to temporary or permanent physical, cognitive, or psychosocial impairment [1,2]. TBIs are an important public health challenge, as they are the most common trauma-related injury leading to disability and death worldwide [3]. The global yearly incidence of TBI is 69 million people, with 1.7 million in the United States [4].

The Glasgow Coma Scale (GCS) is the most widely used tool to classify TBIs into mild, moderate, or severe [5]. Mild TBIs are the most prevalent type [4]. TBIs are associated with a high mortality rate, and multiple researchers have tried to determine the prognosis predictors of it. TBI-related mortality is potentially dependent on age, gender, GCS, the mechanism of injury, the level of consciousness, and the presence of other body injuries, cerebral contusions, epidural hematoma, or skull fractures [6]. The highest incidence of TBIs is among adult males. However, even increasing age is associated with higher mortality in mild TBI cases, the impact of age and gender on TBI-related mortality is still controversial, and most studies report that the severity level is the strongest predictor of mortality [7,8]. One important determinant of mortality in TBI cases is the injury mechanism; while road traffic accidents (RTAs) still cause the most TBIs in most countries, in the United States, falls are the leading cause of TBIs, and firearm-related suicide is the most common cause of TBI-related death [6,9]. Regardless of the severity of TBIs, the distance from the nearest neurosurgery center is proportionally related to the mortality rate [10].

TBI-related mortality is divided into two substantial stages: the primary insult during the time of injury, and the secondary consecutive events and complications [11]. Mortality rates that are directly associated with TBI include death resulting from the initial injury in the acute phase, within hours to days of the incident. Research studies have investigated the predictors for immediate impact of TBI on mortality such as the severity of injury, GCS, neuroimaging findings and type of injury [12,13]. For example, studies have shown that subjects with penetrating TBI show higher mortality rates in comparison to blunt TBI [14]. Direct consequences of TBI are the immediate causes of death in these patients and include intracranial hemorrhage, cerebral edema, and brain herniation [14].

Beyond the acute phase, patients who survive the initial injury show an elevated risk of mortality in comparison to the general population [15]. This increased mortality risk post-TBI stems from multiple factors, including but not limited to neurological sequelae, comorbidities, socioeconomic factors, and lifestyle choices [16]. Longitudinal studies have highlighted a spectrum of complications that contribute to excess mortality, including neurodegenerative disorders such as dementia and Parkinson’s disease, psychiatric conditions such as depression, and substance abuse, epilepsy, and secondary injuries such as falls and substance-related incidents [15,16].

Although the mortality rate due to TBIs is higher in low- and middle-income countries, in 2020, there were more than 64,000 TBI-related deaths in the United States [1,17]. The mortality outcomes of TBIs are usually studied based on the TBI severity classification. According to epidemiological studies, the vast majority of TBI cases are mild and often do not need medical attention [18]. This contrasts moderate–severe TBIs, which are commonly associated with long-term functional impairments and higher rates of mortality [19]. Although severe TBIs contribute to more than two-thirds of TBI-related mortality cases, in a multicenter study, the 5-year mortality risk in mild TBI cases was increased by 47%. Another cohort study confirmed that the presence of moderate TBIs as a result of extracranial injuries doubles the risk of mortality, particularly in elderly patients [10,20]. While many studies have focused on the outcomes of severe TBIs as the main cause of mortality in patients exposed to any form of TBI, this review aims to highlight the predictors of mild and moderate TBIs causing mortality in trauma patients with a discussion about these negative impact factors.

## 2. Methods

Conducting a comprehensive literature search to identify studies relevant to mortality predictors for adult patients with mild–moderate TBI was carried out via searching in the electronic databases including PubMed/MEDLINE, Scopus, PsycINFO, and Google Scholar. Combinations of keywords were used were “traumatic brain injury”, “mild”, “moderate”, “adult”, “mortality”, “predictors” and variations thereof. The search was limited to articles published in the English language and from inception until 2024. The included studies were those that were published in peer-reviewed journals focusing on adult patients (age ≥ 18 years) diagnosed with mild–moderate TBI and reporting risk factors or predictors associated with the outcomes of mortality. Studies that do not provide relevant data on mortality predictors or outcomes, focused solely on severe TBI or pediatric populations, in addition to case reports, conference abstracts or editorials were excluded. The eligibility of the retrieved articles was assessed based on the inclusion and exclusion criteria via screening of the titles and abstracts. Full text articles of potentially relevant studies were then assessed for final inclusion. The results of the included studies were compiled using a narrative synthesis approach because of the expected variety in study designs, populations, and outcomes. Relevant information was employed and arranged based on recurring themes or mortality predictor groups. No personal patient information was accessed or disclosed, and all data were taken from published research.

## 3. TBI Severity Classification

**Figure 1:**
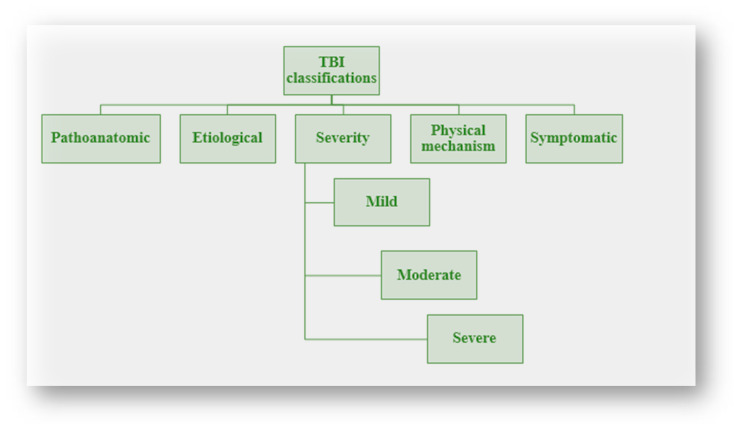
TBI classification. TBIs can be classified based on pathoanatomic, etiology, severity, physical mechanisms, and symptoms.

Historically, multiple ways of classification have been developed to classify clinical diseases. Pathoanatomic classification is used to identify the anatomic locations and features of the trauma. Other ways of classification were developed to achieve different purposes, such as etiological classification, which is used for prevention. For the treatment of specific clinical manifestations, symptomatic classification is used, whereas prognostic classification aims to predict the outcome (Figure 1).

Pathoanatomic classification is considered the most widely used technique [21]. However, this classification faces a special problem because TBIs are usually related to other lesions that differ in location and severity. CT scanning benefits pathoanatomic classification, and CT-based scoring systems have been developed, including the Marshall scoring system in 1992, but this failed to differentiate between the different types of intracranial injuries. A more recent scoring system is the Rotterdam score which is considered better at separating the CT findings and predicting the prognosis of TBIs [22].

**Figure 2:**
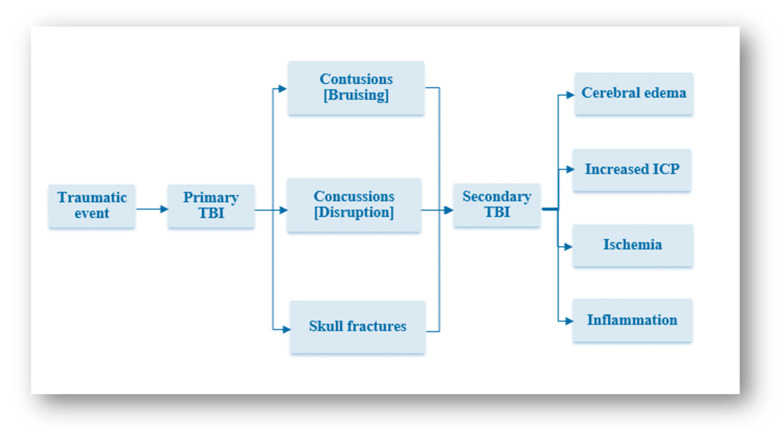
TBI outcomes. Clinical outcomes for TBI patients are classified into primary and secondary. The primary subdivision represents the initial injury that is caused by applied force, and the secondary subdivision represents the delayed consequences which happen because of cellular responses.

Severity and physical causes are still the most frequently observed factors used to classify TBIs [21]. Classifying TBIs depending on the physical cause is insufficient as this may help in predicting the possible outcomes, but it cannot provide enough information about the actual injury [23]. TBIs can also be classified into primary and secondary subdivisions (Figure 2). The primary subdivision represents the initial injury that is caused by applied force, and the secondary subdivision represents the delayed consequences which happen as a result of cellular responses. Numerous subdivisions have been created to describe TBIs in a more detailed manner to precisely predict their outcomes. Primary TBIs could be caused by contact or acceleration–deceleration forces, and secondary TBIs may occur due to different cellular mechanisms at the molecular level; each subdivision has a different prognosis [24]. The severity of TBIs is an important predictor of mortality [25] and the most significant predictor of short-term mortality [7].

The assessment of the severity of TBIs depends on multiple aspects, including the subjective symptoms, mortality risk, required neurosurgical interventions, treatment intensity, hospital duration, quantification of brain tissue injury and biomarkers, the outcome after hospital discharge, functional recovery, independency, and quality of life [26]. Although different severity scales for TBIs have been developed, including the Brussels and Grady coma grades and the Innsbruck, Moscow, Bozza–Marrubini, and Jouvet coma scales, the GCS is the most widely used in clinical practice even after development of the FOUR score scale, which was developed based on the limitations of the GCS [22]. However, other tools like the Galveston Orientation and Amnesia Test (GOAT) or Westmead PTA scale are sometimes used to assess the TBI severity, depending on post-traumatic amnesia (PTA) duration [27,28].

More than 40 years ago, the GCS was developed as a tool for the assessment of the level of consciousness and gives a score between three and fifteen based on the summation of the eye-opening score, out of four; the verbal response score, out of five; and the motor response score, out of six. A higher score is considered better in each subdivision [29]. The GCS divides TBI patients into three groups as follows: mild for patients achieving 13–15, moderate for patients achieving scores of 9–12, and scores of 3–8 represent severe TBIs [5]. Although the GCS is highly predictive of acute mortality and morbidity rates in TBI cases, there are some factors making the GCS untestable, such as in patients who present to a hospital who have already been sedated, paralyzed, or ventilated [30]. The European Brain Injury Consortium reported that the GCS was testable in 77% of patients presenting with a moderate-to-severe TBI [31]. Additionally, other studies have shown that the GCS is a poor reflection of the clinical outcome and lacks reliability as a severity measure [32,33].

Despite PTA being rarely assessed in clinical practice, the recommendation of using PTA as a clinical predictor after a TBI has been mentioned in many studies [34]. Even PTA assessment has a risk of recall bias, as it is often performed in a retrospective way [35]. Many patients who are considered to have a mild TBI based on the GCS are classified as having a severe TBI after PTA assessment, making the concordance between these two scores poor [36,37]. Even though most patients with a mild TBI make a full recovery, there are multiple reasons why a mild TBI can have a severe course or fatal outcome in some cases [38]. This is why a recent study recommended stopping the use of these severity scales and developing a more predictive assessment tool by investing in the available advanced measures of imaging and biomarkers [26].

## 4. Mortality Predictors and Rates of Mild–Moderate TBIs

The understanding and assessment of factors predicting mortality in instances of mild and moderate traumatic brain injuries (TBIs) are essential for improving patients’ outcomes and optimizing the allocation of healthcare resources [39]. The current scientific literature underscores the significance of the GCS as an initial and foundational prognostic measure. Stein and Spettell (1995) demonstrated an inverse association between the initial GCS scores and the mortality risk in mild TBI cases. Nevertheless, recognizing the limitations of the GCS as a singular predictor is imperative, necessitating a comprehensive approach that considers multiple factors [40] (Table 1).

Age consistently arises as a prognostic factor for mortality in cases of mild and moderate traumatic brain injuries (TBIs). There is an unfavorable influence of advanced age on the mortality outcomes among individuals with moderate TBIs. The susceptibility to age-related factors underscores the significance of customized interventions and enhanced surveillance for elderly patients presenting with a mild or moderate TBI [52]. Furthermore, the prognosis of mild and moderate TBIs among patients is complicated by comorbidities and pre-existing medical conditions. A significant correlation between pre-existing conditions and elevated mortality rates within this demographic was observed. Effectively addressing these comorbidities is paramount for the development of good care strategies and accurate risk stratification [53].

Physiological markers, including intracranial pressure (ICP) and cerebral perfusion pressure (CPP), have emerged as crucial predictors of mortality in TBI cases. Elevated ICP is linked to poorer outcomes, underscoring the significance of monitoring and managing intracranial dynamics [54]. Concurrently, maintaining optimal CPP levels has been associated with improved survival rates, as outlined by the Brain Trauma Foundation in 2016. In the realm of imaging, findings such as the extent of contusions, a diffuse axonal injury (DAI), and a midline shift have been correlated with an elevated risk of mortality [55].

Coagulopathy and systemic complications exert a substantial impact on the TBI outcomes. Trauma-induced coagulopathy is notably associated with increased mortality among TBI patients, demanding prompt recognition and intervention [56]. Therefore, the presence and extent of traumatic intracranial hemorrhage, such as epidural hematoma, subdural hematoma, and intraparenchymal bleeding, are correlated with an increased mortality risk. These hemorrhages can cause increased intracranial pressure and subsequent brain damage [57]. Similarly, the presence of skull fractures, particularly in combination with other traumatic brain injury markers, has been associated with higher mortality rates. Skull fractures can result in an increased risk of brain injury and subsequent complications [58]. It must be pointed out that pupillary assessment often forms a part of neurological examinations to predict the outcome. Finding abnormal pupillary response, including fixed and dilated pupils are indicative of a severe traumatic brain injury and associated with an increased risk of mortality [59].

The complexity of TBI-related mortality prediction is further compounded by psychosocial and pre-hospital factors. The presence of pre-existing comorbidities, socioeconomic status, and access to timely pre-hospital care collectively play pivotal roles in determining the outcomes [60]. Additionally, systemic complications like pneumonia, sepsis, and multi-organ failure contribute to a heightened risk of mortality, emphasizing the necessity for comprehensive management strategies [61].

Advancements in neuroimaging techniques provide valuable insights into mortality prediction for individuals with mild or moderate TBI. The prognostic importance of abnormal CT findings in anticipating the mortality outcomes is a strong predictor. The integration of imaging data into predictive models enhances the precision of mortality risk assessment, thereby facilitating well-informed clinical decision making [39,62]. Additionally, recent developments in the exploration of biomarkers offer a promising avenue for refining mortality prediction models in mild and moderate TBI cases. Some serum biomarkers, including glial fibrillary acidic protein (GFAP), have been identified as potential predictors of mortality in cases of CT-negative traumatic brain injuries. The incorporation of biomarkers into clinical practice holds the potential for the early identification of high-risk patients and the formulation of personalized treatment strategies [63,64].

## 5. Biomarkers in Traumatic Brain Injury

Biomarkers of mild and moderate TBIs have garnered significant attention due to their potential utility in diagnosis, prognosis, and treatment monitoring. Recent studies have explored various possible biomarkers involved in the pathophysiology of TBIs, including proteins, microRNAs, and neuroimaging markers. However, challenges persist in standardizing the assay protocols, establishing clinically relevant cutoff values, and accounting for inter-individual variability in biomarker levels. Consequently, the translation of biomarker research into clinical practice requires rigorous validation in large, well-characterized patient cohorts with diverse demographic and clinical profiles [65].

Biomarkers offer objective measures that complement traditional clinical assessments, which often rely on subjective symptom reporting and neuroimaging findings [66]. The identification of reliable biomarkers holds promise for improving the clinical management of patients with mild-to-moderate TBIs by enabling the earlier detection of injury, predicting the outcomes, and guiding therapeutic interventions. Despite extensive research efforts, identifying biomarkers that are both sensitive and specific to mild and moderate TBIs is still in the initial stages. Moreover, the heterogeneity of TBI presentations and the influence of extraneous factors, such as age, sex, and comorbidities, further complicate biomarker validation and interpretation [67].

Protein biomarkers, such as GFAP, tau, and neurofilament light chain (NFL), reflect the extent of neuronal damage and astroglial activation following a TBI [67]. These biomarkers can be detected in blood, cerebrospinal fluid (CSF), and even saliva, offering convenient and non-invasive sampling methods for clinical use [66]. Additionally, high levels of Tau and GFAP in the blood and CSF have been correlated with axonal damage and cognitive impairment in patients with TBI [68]. The TBI severity and outcomes were also proportionally linked to the blood and CSF levels of S100 protein and neuron-specific enolase (NSE) [69,70].

Another biomarker of interest is C-reactive protein (CRP), which is an acute-phase reactant synthesized by the liver in response to tissue injury, inflammation, or infection [71]. Research has demonstrated a correlation between elevated blood CRP levels and increased mortality rates in patients with mild to moderate TBI [62]. Following mild to moderate TBI, CRP levels in blood have been shown to increase, reflecting the systemic inflammatory response elicited by the injury [72]. Higher CRP concentrations within the first 24 to 48 h post-injury have been associated with poorer outcomes and higher mortality rates in patients with mild TBI [73]. Additionally, studies utilizing imaging techniques such as positron emission tomography (PET) have identified the presence of CRP in injured brain regions, suggesting a potential direct role of CRP in neuroinflammation and secondary brain injury mechanisms [74]. Patients who have higher CRP concentration in the brain during the first two weeks of their TBI were associated with higher rates of mortality and disability [75].

One of the other promising biomarkers for TBI diagnosis and prognosis is ubiquitin C-terminal hydrolase L1 (UCHL1) [76]. UCHL1 is an enzyme found abundantly in the neuronal cytoplasm and is involved in the ubiquitin-proteasome system (UPS) regulation [77]. Studies reported that UCHL1 is released into CSF and bloodstream upon TBI. The elevated levels of UCHL1 in CSF and serum were associated with TBI severity and outcomes, suggesting its potential prognostic value [78]. Moreover, the higher serum and CSF levels of UCHL1 in the first six hours of TBI were associated with higher rates of mortality [79]. Despite its role in differentiating TBI from other neurological conditions such as multiple sclerosis and stroke [80], the interpretation of UCHL1 levels must be considered in the context of the clinical presentation and other diagnostic findings as UCHL1 levels are elevated in non-TBI related conditions such as cardiac arrest and spinal cord injury. This limits the use of UCHL1 as an independent biomarker for TBI [79]. 

In addition to protein biomarkers, the emerging evidence suggests that microRNAs, small non-coding RNAs involved in post-transcriptional gene regulation, may serve as sensitive indicators of TBI pathology [66]. MicroRNAs exhibit dynamic expression patterns in response to neuronal injury and have the potential to differentiate between different injury severities and outcomes [81]. Furthermore, advances in neuroimaging techniques, such as diffusion tensor imaging (DTI) and functional magnetic resonance imaging (fMRI), enable the visualization of structural and functional changes in the brain following TBIs. These imaging modalities provide complementary information to biomarker analyses and offer insights into the underlying mechanisms of injury and recovery [66].

## 6. Miscellaneous Factors Affect TBI Outcome

According to the Center for Disease Control and Prevention (CDC), TBIs significantly contribute to annual visits, hospitalizations, and fatalities that happen in emergency departments in the United States [82].

In the United States, the presence of resources, encompassing advanced healthcare facilities and emergency response systems, might contribute to the comparatively diminished mortality rates linked to these injuries. According to the data from the National Center for Health Statistics (NCHS), in 2020, enhancements in trauma care, rehabilitation services, and the availability of neurosurgical interventions have exerted a positive impact on TBI outcomes within the nation. Conversely, nations with fewer resources could encounter distinctive challenges when addressing mortality associated with TBIs. Restricted access to high-quality healthcare, particularly in rural areas, has the potential to impede prompt diagnosis and intervention, thereby exacerbating the severity of TBI outcomes [83]. Countries with lower income levels encounter elevated mortality rates attributed to TBIs, mainly as a result of difficulties in obtaining timely and sufficient medical care. Moreover, a lower socioeconomic status can give rise to disparities in preventive practices, such as the utilization of protective equipment in sports or compliance with safety protocols in workplaces [4]. The World Health Organization (WHO) confirms the connection between socioeconomic factors and strategies for preventing injuries, highlighting the necessity for tailored interventions in settings constrained by limited resources [84].

The cultural context significantly influences the causes and consequences of TBIs on a global scale. Within certain nations, traditional customs or sporting activities can present distinctive hazards for head injuries. Analysis spanning different cultures proposes that cultural gatherings involving physical pursuits lacking sufficient safety measures might elevate the risk of mild and moderate TBIs. In such instances, the importance of awareness campaigns and educational initiatives on injury prevention becomes imperative in mitigating the impact of TBIs on mortality rates [85].

Furthermore, variations in healthcare infrastructure and policies can significantly impact TBI outcomes. Countries with well-established rehabilitation programs and strong social support systems witness improved recovery rates and lower mortality associated with mild and moderate TBIs. In contrast, nations lacking such infrastructure may struggle to provide adequate rehabilitation services, leading to long-term complications and increased mortality rates [39]. Additionally, the role of pre-existing health conditions in conjunction with TBIs cannot be overlooked. Countries with a high prevalence of conditions like hypertension, diabetes, or cardiovascular disease may experience a synergistic effect that exacerbates the mortality risk associated with TBIs [53].

## 7. Conclusions

TBIs are associated with a high mortality rate, and multiple studies have tried to determine its prognosis predictors. While other studies have focused on the outcomes of the severe TBIs as the main cause of mortality in patients, more research work is needed to highlight the mortality predictors of mild and moderate TBIs as a significant cause of mortality in trauma patients. It is important to recognize the necessity of additional recent and rigorous prospective investigations to validate and refine the current predictors. Collaborations on large-scale studies involving multiple centers are indispensable for attaining a more comprehensive understanding of the complex interrelationships among diverse factors that impact mortality outcomes in individuals with a mild or moderate TBI. 

## Figures and Tables

**Table 1:** Overview of the existing literature on predictors of mortality caused by TBIs (2018–2023).

Paper ID	Year	Study Design	Sample Size	Key Finding
Moskowitz, Eliza et al. [41]	2018	A retrospective multi-institutional cohort study	54	Penetrating injury, young age, and higher GCS at admission were associated with lower mortality rates in patients undergoing decompressive craniotomy after TBI
Estraneo, Anna et al. [42]	2018	A prospective observational cohort study	194	In the long term, respiratory complications including infections were the most common cause of death in patients with moderate TBI
Skarupa, David J. et al. [43]	2019	A retrospective observational study	26,871	The incidence and mortality rates for civilian penetrating brain injury have increased over the last 5 years, with self-inflicted injury and prehospital intubation being the most significant predictors of mortality
Gritti, Paolo et al. [44]	2019	A retrospective monocentric study	193	Increasing age is the main acute risk factor and the Oxford Handicap Scale (OHS) is a potential subacute predictor of mortality moderate TBI patients
El-Menyar, Ayman et al. [45]	2020	A retrospective study	654	Any positive result of serum troponin after TBI is associated with higher mortality risk
Chico-Fernández, M. et al. [46]	2020	A retrospective study	465	The observed mortality rate was lower than predicted in very elderly patients admitted to the ICU based on the severity of injury
Gao, Guoyi et al. [3]	2020	A prospective, multicenter, longitudinal, observational study	13,627	The survival outcomes of individuals with TBI were found to be significantly correlated with age, (GCS) score, injury severity score, pupillary light reflex, (CT) findings (specifically, compressed basal cistern and midline shift ≥ 5 mm), the presence of hypoxia, systemic hypotension, altitude exceeding 500 m, and gross domestic product (GDP) per capita
Amare, Abraham Tsedalu et al. [1]	2021	A retrospective cohort study	338	TBI patients with high Glasgow Coma Scale score, bilateral non-reactive pupils, and elevated blood pressure have a lower survival rate
Asim, Mohammad et al. [47]	2021	A retrospective study	1035	GCS scores were lower in patients with TBI having higher Rotterdam or Marshall CT scores, which were associated with higher mortality rates
Kashkoush, Ahmed et al. [48]	2021	A prospective study	695	Hospital mortality was independently associated with GCS scores less than 13, nonreactivity of pupils, escalating Injury Severity Score (ISS), intraventricular hemorrhage, and the need for neurosurgical intervention in patients aged > 79 years old with TBI having subdural hematoma
Estraneo, Anna et al. [49]	2022	A prospective study	143 [traumatic *n* = 55]	In adult patients with prolonged vegetative state/unresponsive wakefulness syndrome (VS/UWS) or minimally conscious state (MCS) after TBI, mortality rate was higher in VS/UWS than MCS especially with older age and lower CRS-R total score
Ghneim, Mira et al. [50]	2022	A prospective observational study	2028	In TBI patients aged ≥ 65 years old, GCS score < 9 was the main predictor of mortality, and relying solely on chronological age might be inadequate for accurately forecasting the mortality outcomes
Réa-Neto, Álvaro et al. [51]	2023	A prospective cohort study	1194	Advanced age, reduced GCS scores, and higher number of concurrent potential secondary injuries are independent predictors of mortality in TBI patients presenting to the ICU

## Data Availability

Not applicable.
